# Mechanistic insights into enhanced drought resistance of modern cultivars and drought-tolerance indicator screening

**DOI:** 10.3389/fpls.2026.1835554

**Published:** 2026-05-15

**Authors:** Yuee Liu, Jinfeng Xing, Tianfang Lv, Wantao Cai, Chunyuan Zhang, Jiuran Zhao

**Affiliations:** Beijing Key Laboratory of Maize DNA Fingerprinting and Molecular Breeding, Maize Research Center, Beijing Academy of Agriculture & Forestry Sciences, Beijing, China

**Keywords:** cultivar, drought resistance, drought resistance index, maize, yield

## Abstract

Cultivar improvement is an important means of enhancing drought resistance in maize. Investigating the reasons for enhanced drought resistance in modern cultivars and screening drought tolerance indicators are of great significance for the breeding of drought-tolerant maize cultivars in China. This study first assess the drought resistance in cultivars from different eras and its influencing factors and then examines the relationship between drought resistance and phenotypic indexes to screen for drought resistance indicators useful for breeders in selecting drought-resistant cultivars. An investigation of drought resistance was performed in a rainout shelter in Hengshui, Hebei, over the 2018 and 2019 growing seasons. The experiment employed a split-split plot design, which comprised nine maize hybrids representing different eras and three water treatment levels (full irrigation, moderate drought, and intense drought). Grain yield and phenotypic indexes of different maize hybrids from different eras (1970s to 2010s) were compared under different water treatments. Maize yield showed a significant linear increase with the year of release (p < 0.01). The maize yield of modern cultivars (Cluster III) under full irrigation, moderate drought, and intense drought was 9.08 t ha^-1^, 7.03 t ha^-1^, and 6.11 t ha^-1^, respectively, which was significantly higher than that of older cultivars (Cluster I, Cluster II). Modern cultivars consistently exhibited superior drought tolerance compared to older cultivars. The enhanced drought resistance and yield in modern maize cultivars were driven primarily by a significant increase in harvest index. Additionally, smaller root size and increased deep roots in modern cultivars were significant contributors to improved drought resistance. Drought had the greatest effect on tasseling to silking interval, the number of ears per hectare, ear height and ear length, which were identified as the drought resistance indicators. Our findings provide valuable insights for breeding and selecting drought-resistant maize cultivars.

## 1 Introduction

In recent years, water scarcity has become a pressing global challenge that poses a critical threat to global food security and agricultural productivity (Langenbrunner, 2021). Over the past decade, drought-induced crop yield losses have amounted to approximately $30 billion (Gupta et al., 2020). Driven by global population growth, agricultural water demand could double by 2050 (FAO, 2011). Conversely, freshwater availability faces a potential 50% reduction, attributed to the increasing frequency and scale of extreme drought events under climate change (Gleick, 2000; Chen et al., 2025). Compared to 2006 levels, global food demand will increase by 50-110% by 2050 (FAO, 2011; Tilman et al., 2011; Tester and Langridge, 2010). Achieving this goal is considerably challenging due to the low efficiency of agricultural water use and the increasingly severe droughts (Otkin et al., 2016, Otkin et al., 2018; Basara et al., 2019; Langenbrunner, 2021; Chen et al., 2025). Improving crop drought resistance is a critical strategy for addressing these challenges (Rickards and Howden, 2012; Howden et al., 2007).

Developing drought-resistant hybrids is an effective way to mitigate drought-related yield losses (Edmeades et al., 2006; Sun et al., 2017; Liu et al., 2023). Under drought conditions, drought-tolerant hybrids can still produce decent yields, ensuring food production (Cooper et al., 2014; Boomsma and Vyn, 2008; Zhao et al., 2018; Sammons et al., 2014; Liu et al., 2021b, Liu et al., 2023). Comparative studies on drought resistance and yield-related traits have been conducted across multiple countries. For example, research in China and the United States has shown that maize yields exhibit a linear increase across successive generations, under both drought and irrigated conditions (Russell, 1991; Castleberry et al., 1984; Barker et al., 2005; Edmeades et al., 2003; Sun et al., 2017; Liu et al., 2023). Compared with traditional cultivars, modern cultivars are better adapted to drought stress (Dwyer et al., 1991; Nissanka et al., 1997; Liu et al., 2023) due to their ability to maintain high ear and grain numbers per ear under drought stress (Liu et al., 2023).

Determining how to breed drought-resistant cultivars and identifying the most effective indices for drought resistance in breeding are important areas of study. Many drought resistance indicators, such as the drought resistance coefficient, sensitivity index, drought damage index, and drought resistance index, have been investigated (Li, 1993; Lan, 1998). Drought tolerance is typically assessed based on phenotypic, physiological, and biochemical responses (Ribaut and Ragot, 2007; Reynolds and Langridge, 2016). Among these, phenotypic responses are particularly important for breeding drought-resistant cultivars. Further research is needed to screen drought-resistant cultivars, identify influencing factors, and establish relationships between phenotypic indicators and drought resistance in maize.

Regarding the research on drought resistance of cultivars from different eras, some of our work has already been published, and the study found that: The maize yield and WUE showed a significant linear increase with the year of release. The yield reduction ratio showed a significant linear decrease from the 1970 s through the 2010s under both moderate water deficit and intense water deficit indicating that modern cultivars had better drought resistance than older ones. The significant increase in the number of ears per hectare and the number of kernels per ear was the main reason for maize yield increase with the year of release from 1970s to 2010s under drought treatments. Under drought stress, the uniformity of yield component decreased significantly compared to the fully-irrigated treatment. In addition, an overly large leaf area was not conducive to drought resistance (Liu et al., 2023). However, the reasons for the enhanced drought resistance of modern cultivars and the associated drought resistance indicators have not yet been reported. Therefore, this study first assess the drought resistance in cultivars from different eras and its influencing factors, and then examines the relationship between drought resistance and phenotypic indexes to screen for drought resistance indicators useful for breeders in selecting drought-resistant cultivars.

## 2 Materials and methods

### 2.1 Experimental site

**Figure 1:**
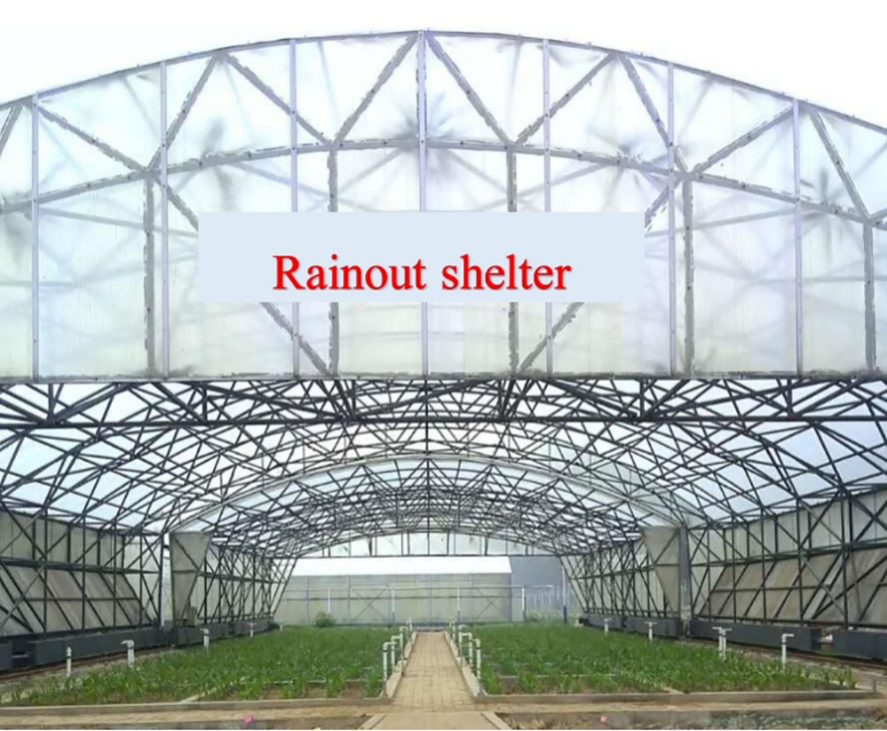
Location of the experiment site.

The experiment was conducted in a rainout shelter in Hengshui (115°40′E, 37°44′N), Hebei Province (**Figure 1**). The shelter completely excluded natural precipitation, allowing precise control of soil moisture through an irrigation system to study crop physiological responses under different water conditions (e.g., establishing drought gradients: mild, moderate, and intense drought). The study was carried out during the 2018 and 2019 growing seasons. The study area featured loam soil under a warm temperate continental monsoon climate. The experimental plots were 2.2 m in length, 3 m in width, and 1.8 m in depth, and each plot included cement lining and waterproofing on all sides and the bottom, with subsequent soil filling.

### 2.2 Experimental design and field management

The experiment included three water treatments (full irrigation, moderate drought, and intense drought), nine maize cultivars, and the experiment was arranged in three replications with a plant density of 60,000 plants ha^−1^. Three water regimes were as follows:

1. Full irrigation: Soil moisture was maintained at 70-80% of the field capacity throughout the maize growth period.
2. Moderate drought: soil moisture was maintained at 50-60% of field water capacity.
3. Intense drought: Water was withheld throughout the entire maize growth cycle.

Nine maize cultivars representing five decades of breeding progress (from the 1970s to the 2010s) were selected. These cultivars were among the most widely grown in China during their respective periods (**Table 1**).

**Table 1:** Maize cultivars used in this study (Liu et al., 2023).

Genotypes name	Pedigree	Year of release/use	Institution developing the hybrid	Decade of cultivation
Zhongdan2	Mo17×Zi330	1975	Chinese Academy of Agricultural Sciences., Beijing, China	1970s
Danyu13	Mo17×E28	1986	Dandong Academy of Agricultural Sciences of Liaoning Province, Dandong, China	1980s
Yedan13	Ye478×Dan340	1992	Laizhou Academy of Agricultural Sciences of Shandong Province, Laizhou, China	1990s
Nongda108	X178×HuangC	1997	China Agricultural University, China	1990s
Zhengdan958	Zheng58×Chang7-2	2000	Luohe Academy of Agricultural Sciences of Henan Province, Luohe, China	2000s
Xundan20	Xun92-8×Xun9058	2003	Agricultural Science Research Institute of Xun County, Henan Province, China	2000s
Xianyu335	PH6WC×PH4CV	2004	The Tieling Pioneer limited company, Tieling, China	2000s
Jingke968	Jing724×Jing92	2011	Maize Research Center, Beijing Academy of Agriculture & Forestry Sciences, Beijing, China	2010s
Jingnongke728	JingMC01×Jing2416	2017	Maize Research Center, Beijing Academy of Agriculture & Forestry Sciences, Beijing, China	2010s

The experiment followed a split-split plot design, with water treatments as main plots and maize cultivars as subplots. Seeds were sown manually with double seeds per hole, with five rows per plot, a planting depth of 5 cm, and a row spacing of 60 cm. Planting took place on June 15, and harvesting occurred on October 15. Fertilizer application rates were established based on pre-plant soil analysis of N, P, and K:

Nitrogen (N): 250 kg ha^-1^.
Phosphorus (as P_2_O_5_): 150 kg ha^-1^.
Potassium (as K_2_O): 150 kg ha^-1^.

### 2.3 Measurements

Key phenological events were recorded, including sowing, silking, maturity, and harvest. The silking standard is the time when 60% of plants showing silk emergence. Physiological maturity was the day when the black layer appeared on kernels. Key phenological events measurements followed the standardized methods described by Shi et al. (2006).

Leaf Area Measurement: The leaf area of all expanded leaves on maize plants was measured at silking by recording leaf length (L) and maximum leaf width (W). The leaf area (LA) was calculated using the equation as follows:


LA = 0.75×L×W

Root Sampling: To investigate the effect of drought on maize roots, this study selected maize cultivars from different eras with large differences in drought resistance to observe root changes. At the silking stage, we collected maize root samples under full-irrigation and moderate drought treatments. For each treatment, three plants were selected; the above-ground portions were cut manually, and roots were excavated layer by layer using the soil profile method (Sattelmacher et al., 1993). Sampling was conducted at depths of 0–10 cm, 10–20 cm, 20–40 cm, and 40–60 cm, with a sampling area of 28 cm × 60 cm per layer. The collected root-soil mixtures from each layer were placed in nylon mesh bags. Roots were carefully hand-picked, rinsed with water, and then oven-dried at 85 °C to constant weight to determine root dry weight. The corresponding shoot dry weight was measured simultaneously.

Photosynthetic Rate Measurement: Photosynthetic rate was measured on ear leaves at the silking stage using a Li-COR 6400 portable photosynthesis system (LI-COR, Inc., Lincoln, NE, USA). Three biological replicates were measured for each treatment under controlled conditions: The system was operated at a photosynthetic photon flux density of 2,000 μmol m^-^² s^-1^, with the CO_2_ concentration controlled at 500 μmol mol^-1^ (Liu et al., 2020).

Yield and Kernel Analysis: After maize reached physiological maturity, the central two rows (2.2 m row length) of each plot were selected for yield measurement. Ten ears were then sampled to determine kernel rows per ear, kernels per row, ear length, ear diameter, and barren tip length. The sampled ears were subsequently hand-threshed to measure kernel weight and grain moisture content. Kernel moisture content was measured using a portable moisture meter (Model PM8188, Kett, Japan). Grain yield and 1000-kernel weight were then expressed on a 14.0% moisture basis.

### 2.4 Statistical analysis

Data analysis and the production of tables and figures were performed using Microsoft Excel 2017. Regressions between the maize yield, above-ground biomass, harvest index, net photosynthetic rate and year of release were plotted. The year of release was independent variables and the maize grain yield, above-ground biomass, harvest index, net photosynthetic rate were dependent variables. The relationship between the maize yield and different phenotype indices was analyzed. The differences of the maize grain yield, above-ground biomass, harvest index, root weight and net photosynthetic rate between different treatments were tested by using three-way analysis of variance (ANOVA) with the Duncan test at a 5% significance level. Multiple stepwise regressions and correlations between the maize yield and different phenotype indices were performed. All data analysis was conducted by using SAS 9.4.

## 3 Results

### 3.1 Evolution of drought tolerance in maize cultivars (1970s-2010s) under different water conditions

**Figure 2:**
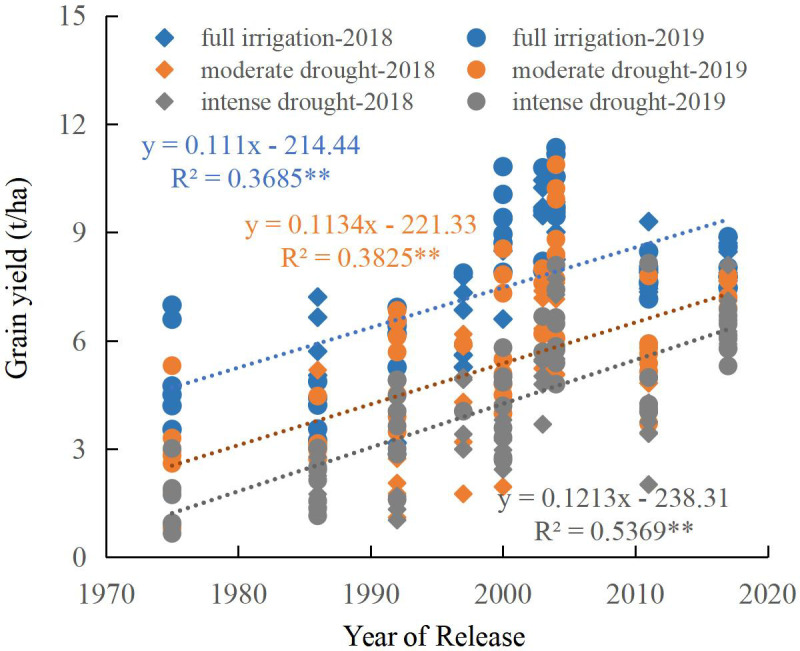
Regression of grain yield of cultivars as a function of the year of release (Liu et al., 2023). ** indicate that the regression correlation is significant at the P < 0.01 level.

**Figure 3:**
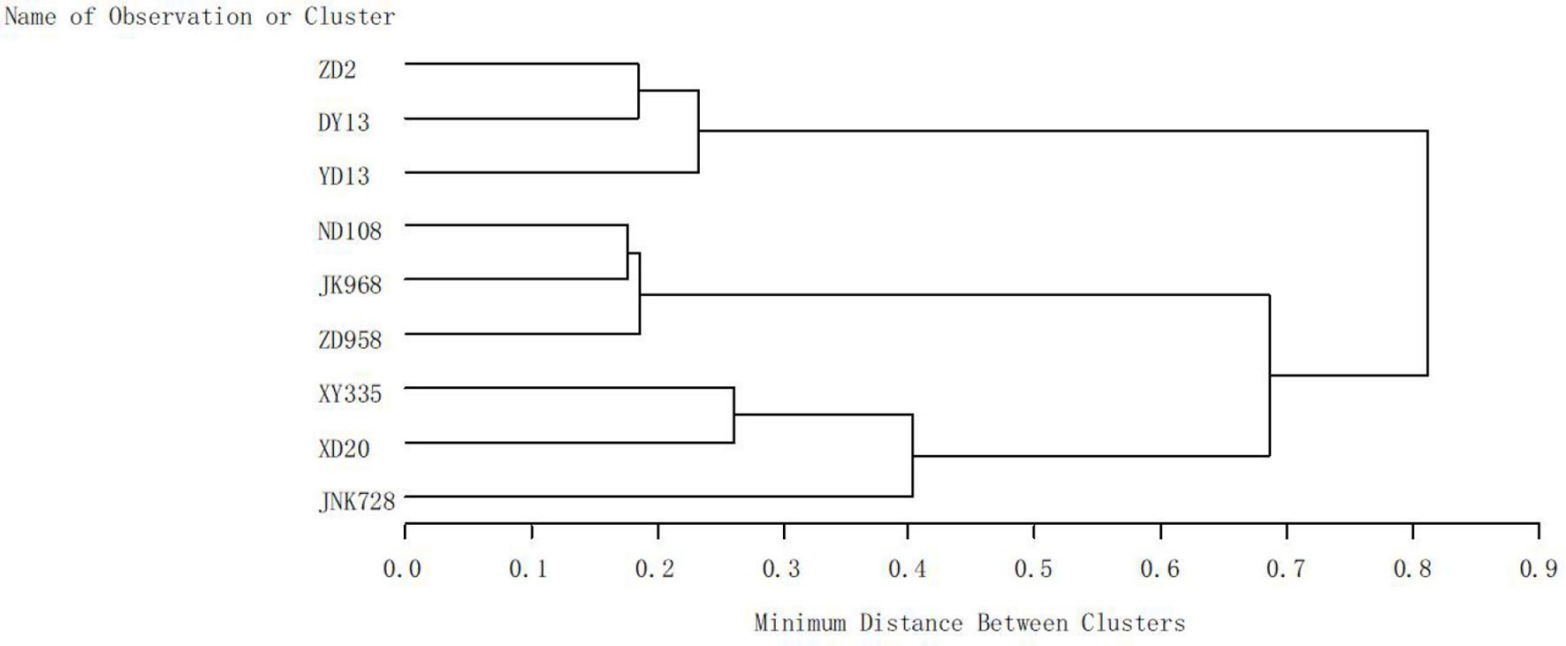
Cluster analysis of drought resistance across different maize cultivars. The clustering analysis was performed using the single linkage method (nearest neighbor clustering) based on yield of varieties from different eras under different water treatments.

With the release year, maize yield increased significantly (*p* < 0.01). Analysis revealed a significant linear upward trend from the 1970s to the 2010s across all water regimes, including full irrigation and moderate and intense drought stress (**Figure 2**). Modern cultivars exhibited strong drought resistance. Through cluster analysis of drought resistance of maize cultivars, three categories were identified, with significant differences in maize yield among them (**Figure 3**; **Table 2**). Cluster I included ZD2, DY13, and YD13, with yields of 4.83 t ha^-1^, 3.24 t ha^-1^, and 2.04 t ha^-1^ under full irrigation, moderate drought, and intense drought, respectively. Cluster II included ND108, ZD958, and JK968, with yields of 7.85 t ha^-1^, 5.14 t ha^-1^, and 3.99 t ha^-1^ under full irrigation, moderate drought, and intense drought, respectively. Cluster III included XD20, XY335, and JNK728, with yields of 9.08 t ha^-1^, 7.03 t ha^-1^, and 6.11 t ha^-1^ under full irrigation as well as under both moderate and intense drought (**Table 2**). Significant yield differences were observed among Clusters I, II, and III under all water conditions (full irrigation, moderate drought, and intense drought), with yield following the order: Cluster III> Cluster II > Cluster I. Within each cluster, yield also differed significantly across water conditions, following the order: full irrigation > moderate drought > intense drought (**Table 2**).

**Table 2:** Changes in maize yield (t/ha) under different classifications and water conditions.

Category	Hybrids	Full irrigation	Moderate water deficit	Severe water deficit
I	ZD2, DY13, YD13	4.83d	3.24e	2.04f
II	ND108, ZD958, JK968	7.85b	5.14d	3.99e
III	XD20, XY335, JNK728	9.08a	7.03b	6.11c

Values marked with different lowercase letters (a or b) are significantly different at the P < 0.05 level.

### 3.2 Trends in above-ground biomass from the 1970s to the 2010s under different water regimes

**Figure 4:**
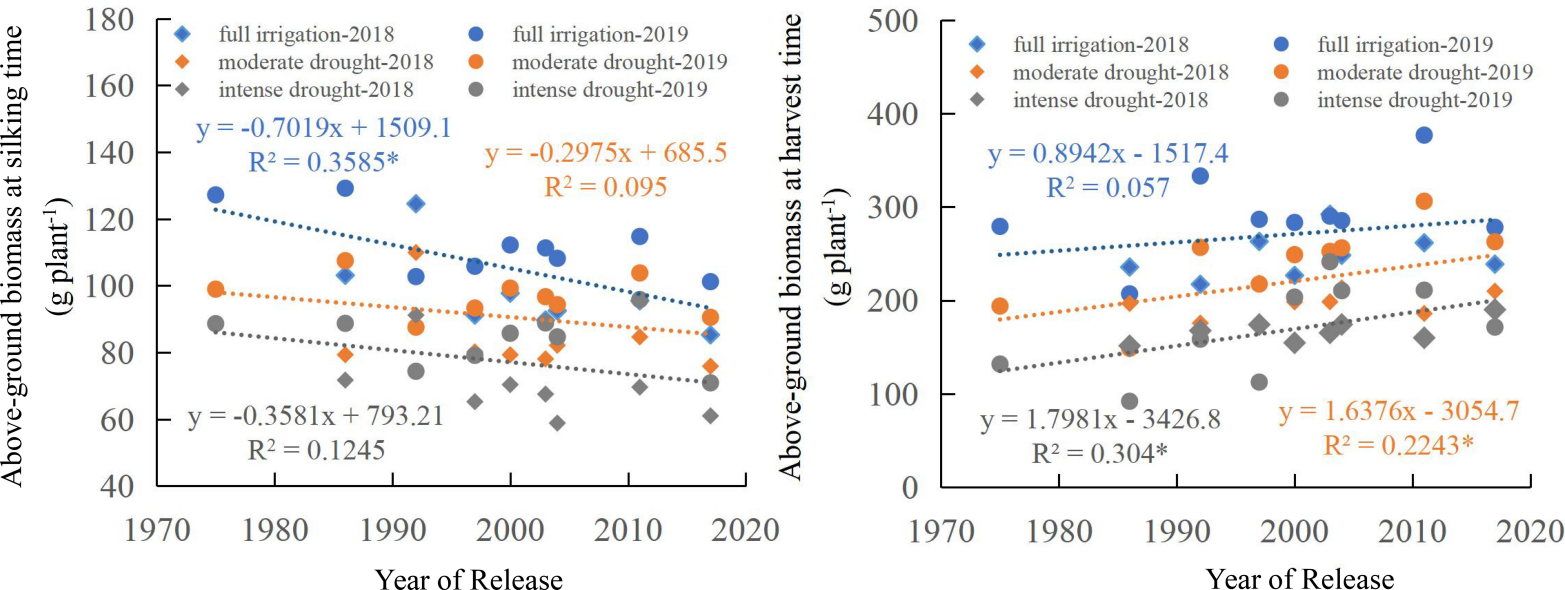
Regression of above-ground biomass as a function of the year of release. * indicate that the regression correlation is significant at the P < 0.05 level.

**Figure 5:**
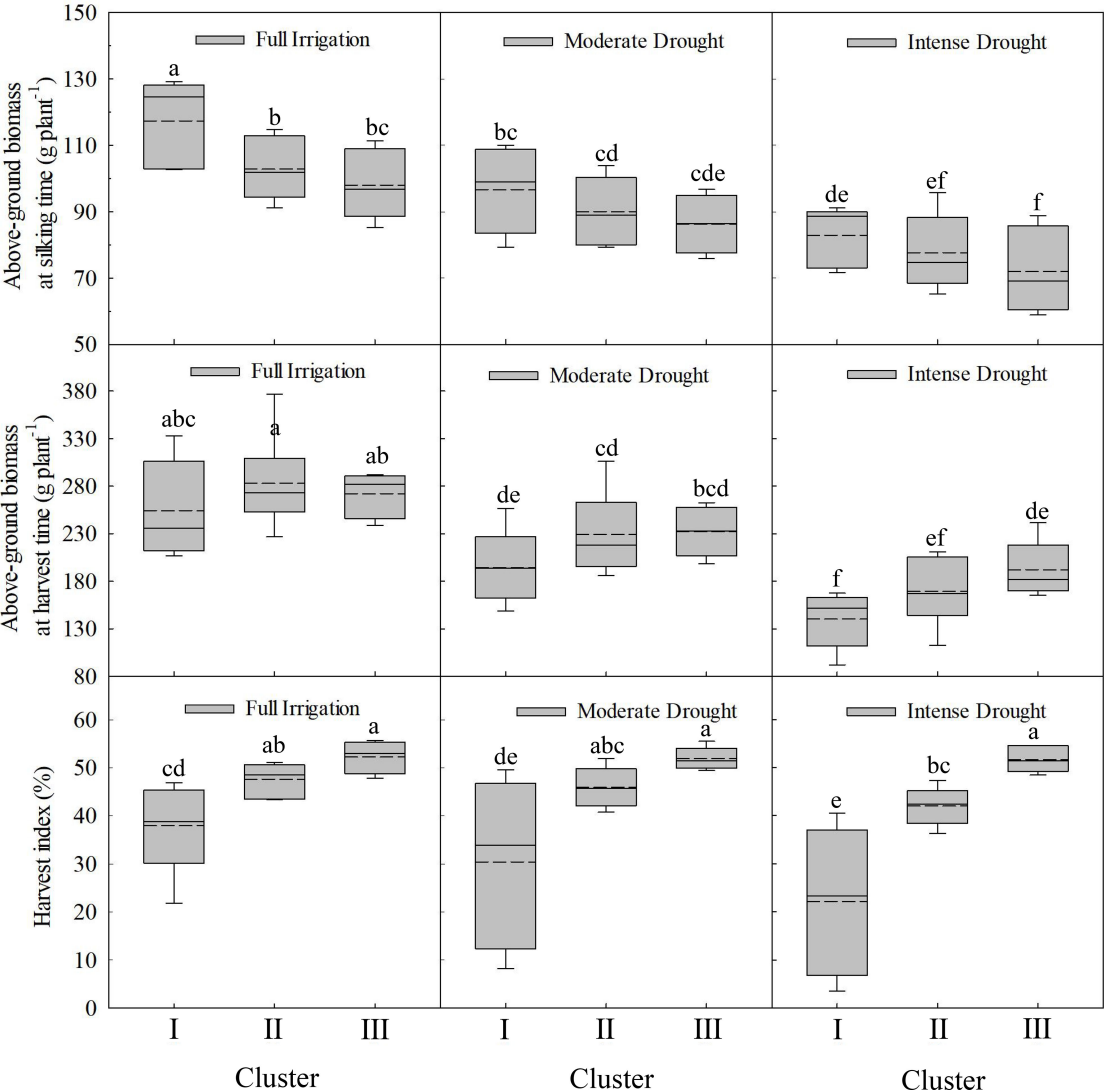
Changes in above-ground biomass and harvest index across different classifications and water conditions. Treatments marked with different lowercase letters (a or b) are significantly different at the P < 0.05 level.

The above-ground biomass of maize cultivars at silking time under full irrigation decreased significantly over time (as newer cultivars were released) (*p* < 0.05). Under moderate and intense drought treatments, from the 1970s to the 2010s, the above-ground biomass at silking stage showed a decreasing trend, though not at a statistically significant level (**Figure 4**). Declines in average above-ground biomass at silking were observed under all water regimes: 0.70 g plant^-1^yr^-^¹ (full irrigation), 0.30 g plant^-1^yr^-1^ (moderate drought), and 0.36 g plant^-1^yr^-1^ (intense drought). Analysis of above-ground biomass at silking time across different categories (Cluster I, II, III) revealed that as water stress intensified, maize above-ground biomass significantly decreased. The above-ground biomass of Cluster I, II, and III showed a gradually declining trend, with modern cultivars exhibiting the lowest above-ground biomass (**Figure 5**). Under both full irrigation and intense drought conditions, there were significant differences (*p* < 0.05) in above-ground biomass at the silking time among the cluster memberships (I, II, III). However, under moderate drought conditions, no significant differences in silking-stage biomass were observed among the different cluster memberships (I, II, III).

At harvest time, a significant linear upward trend in maize above-ground biomass was identified from the 1970s to the 2010s under drought conditions (both moderate and intense). However, no significant linear relationship was found under full irrigation (**Figure 4**). The annual increase in average above-ground biomass at harvest was quantified as 0.89 g plant^-^¹yr^-1^(full irrigation), 1.64 g plant^-1^yr^-1^ (moderate drought), and 1.80 g plant^-1^yr^-1^(intense drought). Analysis of above-ground biomass at harvest across the three categories (Cluster I, II, III) revealed that as water stress intensified, maize above-ground biomass decreased significantly. Under all water conditions, the above-ground biomass of Cluster I, II, and III showed a gradual increase, with modern cultivars exhibiting the highest above-ground biomass at harvest. This advantage was particularly pronounced under drought conditions. Under intense drought, significant differences (*p* < 0.05) in above-ground biomass at harvest time were observed among the three categories (Cluster I, II, and III). However, under full irrigation and moderate drought, no significant differences in harvest-time above-ground biomass were observed among the three categories (Cluster I, II, III) (**Figure 5**).

**Figure 6:**
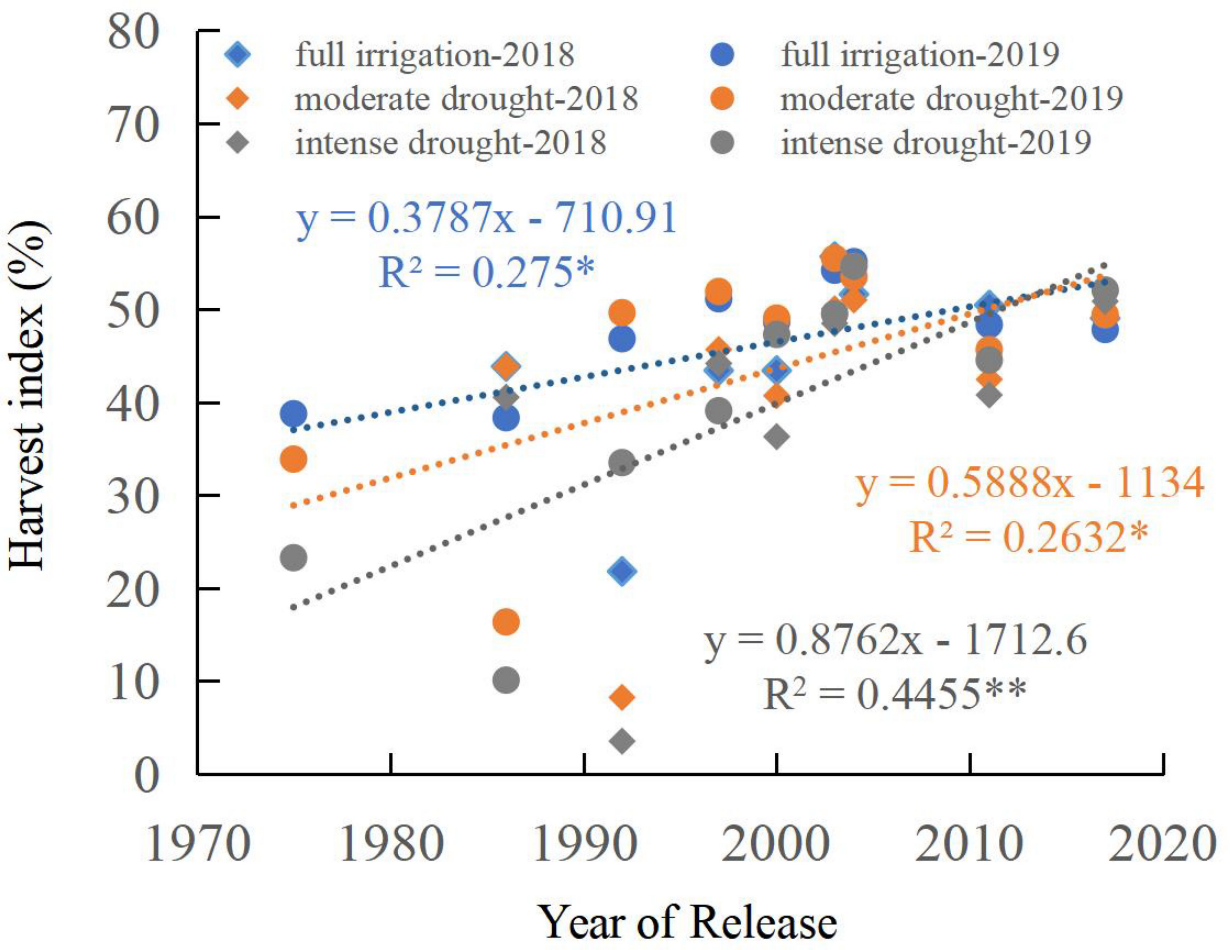
Regression of harvest index as a function of the year of release. * or ** indicate that the regression correlation is significant at the P < 0.05 or P < 0.01 level, respectively.

The harvest index of maize cultivars showed a significant increase (*p* < 0.05) with the year of release across all water regimes (*p* < 0.05). A significant linear upward trend in the harvest index was observed from the 1970s to the 2010s under full irrigation as well as under moderate and intense drought stress (**Figure 6**). The annual increase in the average harvest index was 0.38% (full irrigation), 0.59% (moderate drought), and 0.88% (intense drought). A significant increase in harvest index was observed under full irrigation relative to drought stress across the studied eras (1970s–2010s). The gap in harvest index between fully irrigated and drought conditions (both moderate and intense) significantly narrowed with the year of cultivar release (**Figure 6**). Analysis of harvest index among different categories revealed: significant differences were observed in harvest index across different categories (Clusters I, II, and III) under all water conditions, from full irrigation to moderate and intense drought. The harvest index increased progressively from Cluster I to Cluster III across all water regimes. Particularly noteworthy was Cluster III, which showed higher harvest index values under drought stress (**Figure 5**).

### 3.3 Maize root weight changes from the 1970s to the 2010s under different irrigation regimes

**Figure 7:**
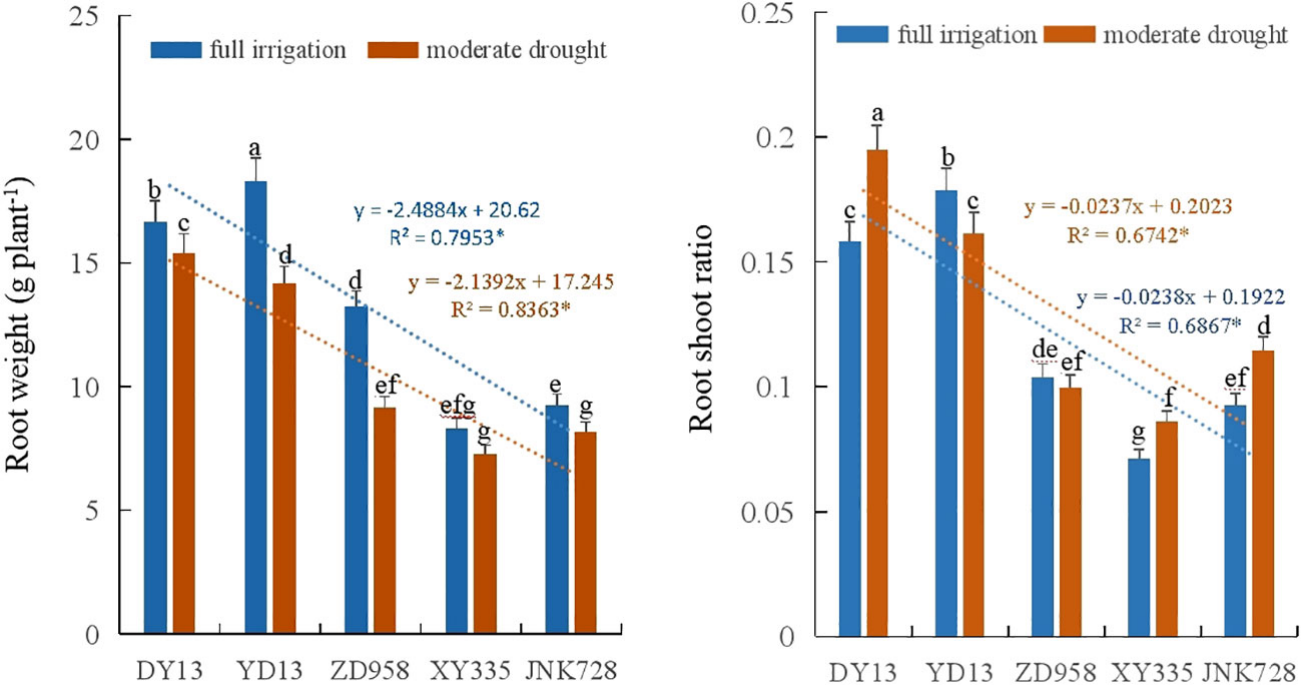
Changes in root weight and root-shoot ratio across different maize cultivars. Treatments marked with different lowercase letters (a or b) are significantly different at the P < 0.05 level. * indicate that the regression correlation is significant at the P < 0.05 level.

Significant variations in root dry weight were observed among maize cultivars (*p* < 0.05). With the breeding and release of new cultivars, root dry weight showed a significant decline under both full irrigation and moderate drought conditions (*p* < 0.05). Drought stress significantly impaired root growth, resulting in a markedly lower root dry weight compared to the fully irrigated treatment (*p* < 0.05). Regarding the root-to-shoot ratio, older maize cultivars exhibited a significantly higher value than their modern counterparts (**Figure 7**). As cultivars were modernized, a significant decrease in the root-to-shoot ratio was consistently observed under both full irrigation and moderate drought stress (*p* < 0.05).

**Figure 8:**
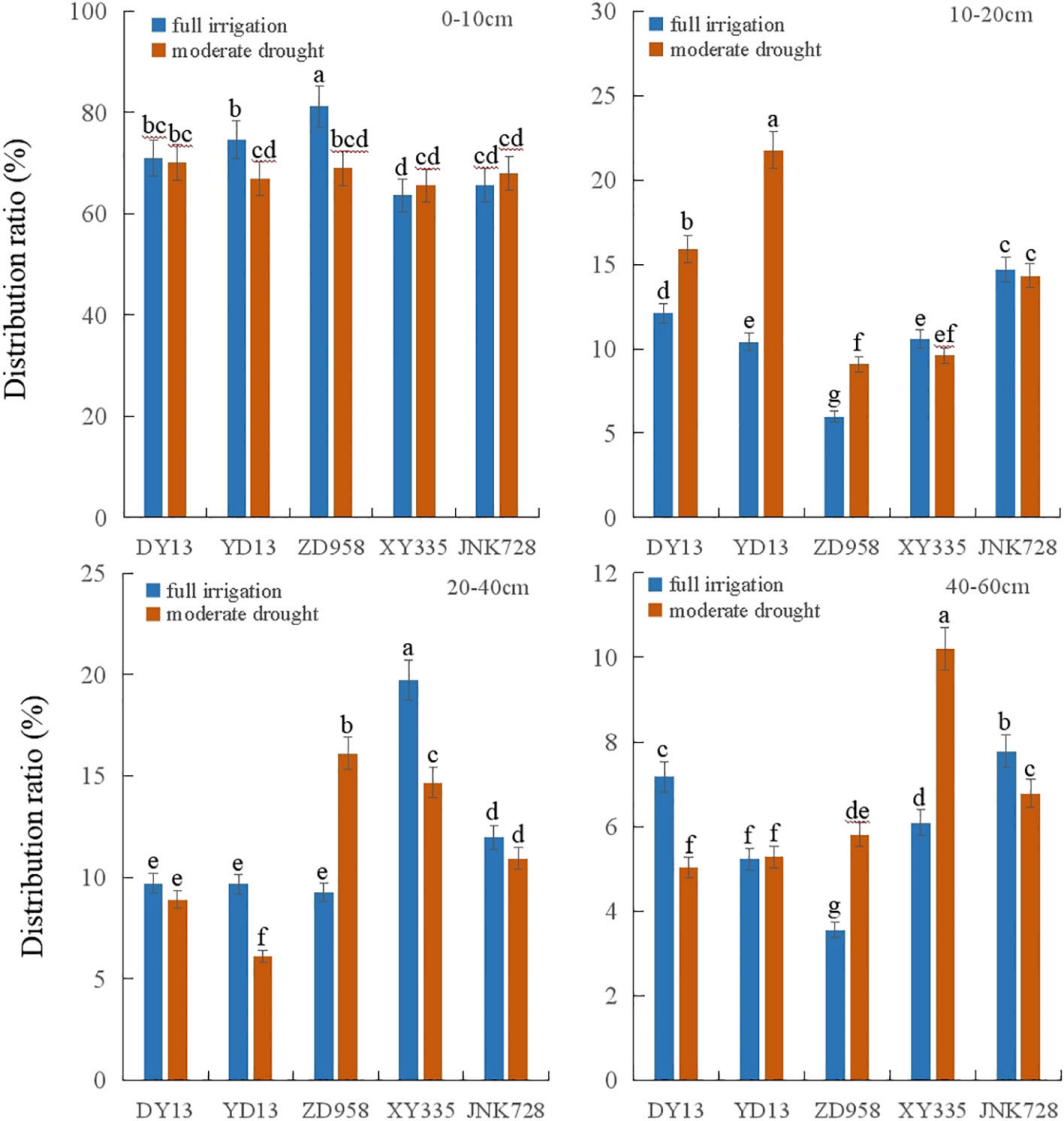
Changes in root distribution ratio of different maize varieties across different soil layers. Treatments marked with different lowercase letters (a or b) are significantly different at the P < 0.05 level.

Analysis of maize root distribution ratio revealed that the root distribution ratio in 0–10 cm soil layer of DY13, YD13, ZD958, XY335, and JNK728 was 71.03%, 74.68%, 81.18%, 63.59%, and 65.57% under full irrigation and 70.14%, 66.87%, 68.99%, 65.53%, and 67.96% under moderate drought stress, respectively. The 0-10 cm distribution ratio of older maize cultivars, such as DY13, YD13, and ZD958, under full irrigation was significantly higher than that of newer cultivars (XY335, JNK728). Under drought stress, the 0-10 cm root distribution ratio of DY13, YD13, and ZD958 decreased compared with the full irrigation treatment, with declines in YD13 and ZD958 reaching significance (*p* < 0.05). In contrast, the 0-10 cm root distribution ratio of XY335 and JNK728 increased under drought stress, though the increases were not statistically significant (**Figure 8**).

Analysis of maize root distribution in the 10-20 cm soil layer revealed that, compared to the full irrigation treatment, the proportion of roots distributed in the 10–20 cm layer significantly increased for DY13, YD13, and ZD958 (*p* < 0.05). In contrast, the proportion of roots in the 10-20 cm layer decreased for XY335 and JNK728, but the reductions were not statistically significant (**Figure 8**).

Analysis of the root distribution ratio in the 20–40 cm soil layer showed that, compared with full irrigation, drought stress decreased the root distribution ratio in this layer for DY13, YD13, XY335, and JNK728, with YD13 and XY335 reaching statistical significance. In contrast, ZD958 showed a significant increase in root distribution ratio in this layer (*p* < 0.05). Analysis of differences in root distribution ratio among maize cultivars revealed that under both full irrigation and drought conditions, the root distribution ratio of DY13 and YD13 was significantly lower than that of XY335 and JNK728, with the difference particularly pronounced under drought stress (*p* < 0.05).

Analysis of the root distribution ratio in the 40–60 cm soil layer revealed that under drought conditions, the root distribution ratio of ZD958 and XY335 increased significantly compared with full irrigation. At the same time, that of DY13 and JNK728 decreased significantly (*p* < 0.05), with no significant change observed in YD13. Analysis of differences in root distribution ratios among maize cultivars showed that under drought conditions, the root distribution ratio in the 40–60 cm layer of modern cultivars XY335 and JNK728 was significantly higher than that of the older cultivars DY13, YD13, and ZD958 (**Figure 8**).

### 3.4 The maize net photosynthetic rate change from the 1970s to the 2010s under different water regimes

**Figure 9:**
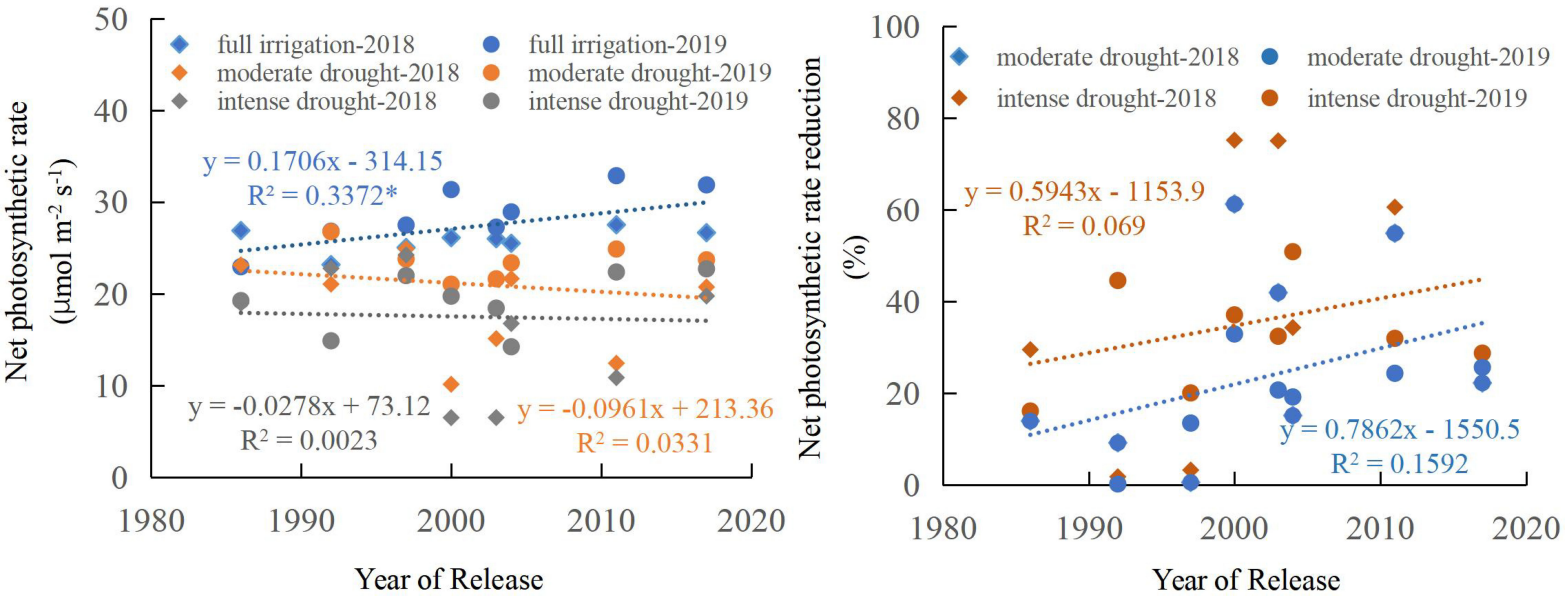
Regression of net photosynthetic rate and reduction of net photosynthetic rate as a function of the year of release. * indicate that the regression correlation is significant at the P < 0.05. Net photosynthetic rate reduction relative to well-watered conditions (%).

A significant linear increase in maize net photosynthetic rate from the 1970s to the 2010s was observed under fully irrigated treatments (*p* < 0.05) (**Figure 9**). The average net photosynthetic rate increased with the year of release, at a rate of 0.17 μmol m^-2^s^-1^yr^-1^ under full irrigation. Maize net photosynthetic rate declined significantly under both drought stress levels relative to well-watered conditions. Over time, the maize net photosynthetic rate declined under moderate and intense drought conditions, but no significant relationships were found. During moderate and intense drought, the reduction in maize net photosynthetic rate increased with the year of cultivar release, although the relationship was not statistically significant (**Figure 9**).

**Figure 10:**
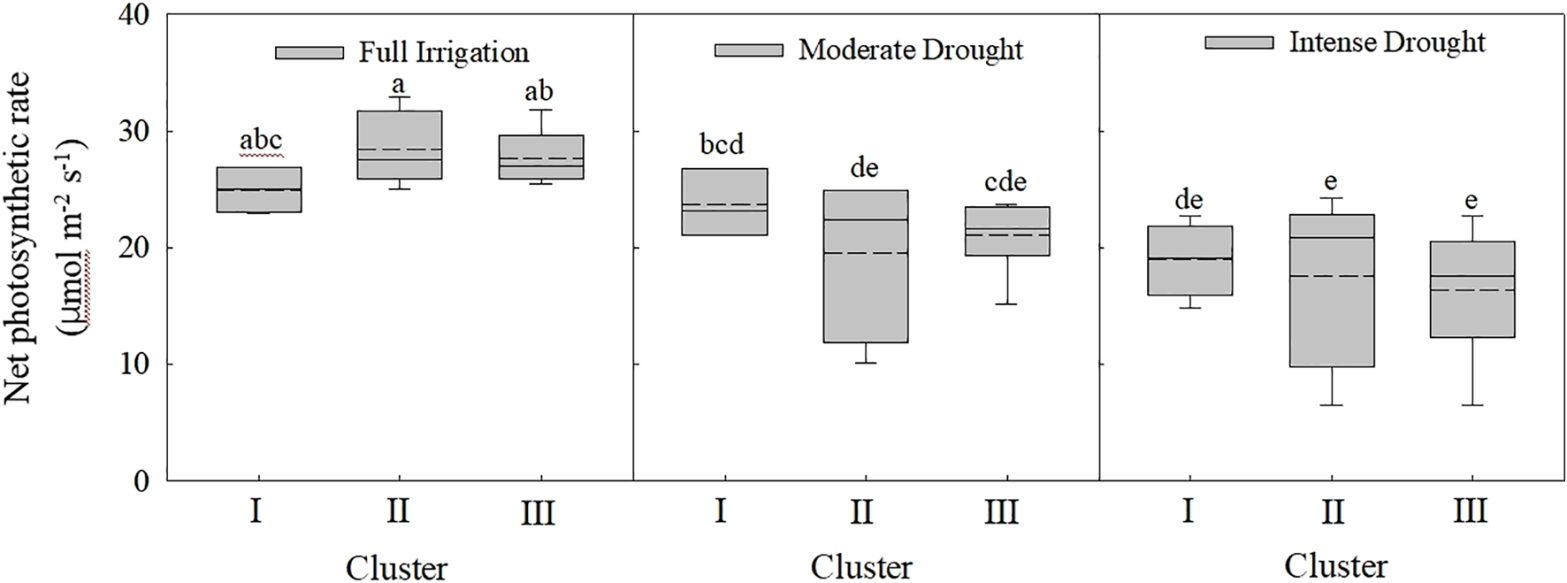
Changes in net photosynthetic rate under different classifications and water conditions. Treatments marked with different lowercase letters (a or b) are significantly different at the P < 0.05 level.

Analysis of net photosynthetic rates across different maize categories (Cluster I, II, and III) revealed that under full irrigation conditions, the rates of Cluster II and III were higher than that of Cluster I, but the differences were not statistically significant. Under moderate and intense drought conditions, the net photosynthetic rate showed a gradual decreasing trend from Cluster I to Cluster III, though none of these changes reached statistical significance. Analysis of differences in net photosynthetic rates across varying water conditions indicated that under full irrigation, net photosynthetic rates were significantly higher than under moderate and intense drought conditions. However, there was no significant difference in net photosynthetic rate between moderate and severe drought conditions (**Figure 10**).

### 3.5 Screening the drought resistance index of maize

Correlation analysis between maize yield and phenotypic indices indicated significant relationships between maize yield (Y) and tasseling to silking interval (X1), ear number per hectare (X2), ear kernel number (X3), 1000-kernel weight (X4), SPAD at silking (X7), SPAD at 30 days after silking (X8), ear length (X9), ear diameter (X10), plant height (X12), and ear height (X13). As ear number per hectare (X2), ear kernel number (X3), 1000-kernel weight (X4), SPAD at silking (X7), SPAD at 30 days after silking (X8), ear length (X9), ear diameter (X10), plant height (X12), and ear height (X13) increased, maize yield increased significantly. However, as the tasseling to silking interval (X1) increased, maize yield decreased significantly. The correlation coefficients between maize yield and phenotypic indices ranked in descending order as follows: ear number per hectare (X2) > tasseling to silking interval (X1) > ear kernel number (X3) > ear diameter (X10) > SPAD at 30 days after silking (X8) > ear length (X9) > SPAD at silking (X7) > ear height (X13) > plant height (X12). No significant correlations were found between maize yield (Y) and leaf area per plant at silking (X5), leaf area per plant at 30 days after silking (X6), or barren ear tip length (X11) (**Table 3**).

**Table 3:** Correlation analysis between maize yield and different phenotype indices.

	Y	X1	X2	X3	X4	X5	X6	X7	X8	X9	X10	X11	X12	X13
Y	1.00000	**-0.75413** <.0001	**0.80283** <.0001	**0.60225** 0.0009	0.39700 0.0403	0.16037 0.4243	0.25836 0.1932	**0.53726** 0.0039	**0.58549** 0.0013	**0.58501** 0.0014	**0.60616** 0.0008	-0.19132 0.3391	0.43636 0.0229	0.45890 0.0161
X1		1.00000	**-0.63611** **0.0004**	**-0.62083** 0.0005	-0.19857 0.3208	-0.11009 0.5846	-0.15203 0.4491	-0.18701 0.3503	-0.14664 0.4655	-0.07901 0.6953	-0.41492 0.0314	0.44110 0.0213	-0.14264 0.4778	-0.10082 0.6168
X2			1.00000	**0.57677** 0.0016	0.38660 0.0464	0.08090 0.6883	0.20920 0.2950	**0.53117** 0.0044	0.51896 0.4655	**0.53046** 0.0044	**0.56809** 0.0020	0.08252 0.6824	0.35628 0.0681	0.17874 0.3724
X3				1.00000	0.23277 0.2426	**0.51651** 0.0058	**0.58933** 0.0012	0.30961 0.1161	0.29014 0.1421	0.18227 0.3628	**0.60476** 0.0008	-0.39962 0.0389	-0.02969 0.8831	0.20516 0.3046
X4					1.00000	0.09064 0.6530	0.20739 0.2993	**0.69935** <.0001	**0.55784** 0.0025	0.46876 0.0137	0.11822 0.5570	0.26511 0.1814	**0.82550** <.0001	0.22136 0.2672
X5						1.00000	**0.96364** <.0001	0.26554 0.1807	0.30234 0.1253	0.20250 0.3111	0.34485 0.0781	-0.42410 0.0275	-0.01946 0.9233	0.41199 0.0327
X6							1.00000	0.37453 0.0543	0.43144 0.0246	0.26086 0.1888	0.45165 0.0180	-0.35208 0.0717	0.03739 0.8531	0.40172 0.0378
X7								1.00000	**0.91707** <.0001	**0.80012** <.0001	0.50602 0.0071	0.33606 0.0866	**0.54924** 0.0030	0.30843 0.1175
X8									1.00000	**0.84316** <.0001	**0.61406** 0.0007	0.33058 0.0921	0.45694 0.0166	0.32851 0.0943
X9										1.00000	0.45098 0.0182	0.42590 0.0268	**0.53672** 0.0039	0.40566 0.0358
X10											1.00000	-0.20641 0.3016	-0.05995 0.7664	0.20000 0.3172
X11												1.00000	0.25692 0.1958	-0.32376 0.0995
X12													1.00000	0.41665 0.0306
X13														1.00000

Y, X1, X2, X3, X4, X5, X6, X7, X8, X9, X10, X11, X12, and X13 represent maize yield, tasseling-to-silking interval, ear number per hectare, ear kernel number, 1000-kernel weight, leaf area per plant at silking, leaf area per plant at 30 days after silking, SPAD at silking, SPAD at 30 days after silking, ear length, ear diameter, barren ear tip length, plant height, and ear height, respectively.

Note: To facilitate the interpretation of the correlation matrix, values with r > 0.5 in Table 3 are shown in bold font.

To further screen the drought resistance indicators of maize cultivars, a stepwise regression analysis was conducted between maize yield and 13 phenotypic traits (**Table 4**). Four indicators significantly correlated with maize yield were selected, namely ear number per hectare (X2), ear height (X13), tasseling to silking interval (X1), and ear length (X9).

**Table 4:** Multivariate regression model between maize yield and different phenotype indices.

Variable entered	Number Vars In	Partial R-square	Model R-square	F Value	Pr>F
X2	1	0.6445	0.6445	45.33	<.0001
X13	2	0.1028	0.7473	9.76	0.0046
X1	3	0.1030	0.8503	15.83	0.0006
X9	4	0.0525	0.9028	11.89	0.0023

## 4 Discussion

### 4.1 Maize cultivars drought resistance differences under different water regimes

Water scarcity has emerged as a critical barrier, hindering further improvements in crop yields (Huang and Li, 2010). Previous research has extensively examined crop water requirements (Liu et al., 2021b; Gardiol et al., 2003) and has evaluated the effects of drought stress on maize development (Almaraz et al., 2008). Research has demonstrated that genetic improvement significantly enhances maize yield (Li et al., 2015; Long et al., 2010; Liu et al., 2021a; Mandić et al., 2016), contributing up to 50% of yield gains (Duvick, 2005; Ma et al., 2015; Ci et al., 2011). Developing drought-tolerant cultivars is essential for maintaining high yields under water-limited conditions. By enhancing inherent drought tolerance, cultivar development helps maintain high yields under water scarcity (Mounce et al., 2016; Edmeades et al., 2006; Sun et al., 2017), thereby stabilizing overall crop productivity under stress (Cooper et al., 2014; Zhao et al., 2018; Sammons et al., 2014; Liu et al., 2023). Consistent with existing research, the current study found significant differences between maize cultivars. Through cluster analysis of drought resistance among different maize cultivars, three categories were identified, with modern cultivars exhibiting the highest yields under both irrigation and drought conditions (**Figure 3**, **Table 2**). Modern cultivars have stronger drought resistance than older ones, which contrasts previous studies suggesting that maize yield sensitivity to drought stress increased from the 1980s through the 2000s (Meng et al., 2016; Lobell et al., 2014).

### 4.2 Key factors influencing maize drought resistance (1970s-2010s)

Previous research observed an increase in maize yield under drought conditions between the 1970s and 2010s, driven predominantly by a prominent rise in both the number of ears per hectare and the kernel count per ear (Liu et al., 2023). In this study, the above-ground biomass of the maize cultivars at silking under full irrigation decreased significantly from the 1970s to the 2010s (*p* < 0.05). Still, no significant relationship was found under moderate and intense drought conditions (**Figure 4**). At harvest time, a significant linear upward trend in maize above-ground biomass was identified from the 1970s to the 2010s under drought conditions (both moderate and intense). However, no significant linear relationship was found under full irrigation (**Figure 4**). Examination of maize cultivars from the 1970s to the 2010s revealed a significant linear increase in harvest index across all water regimes (*p* < 0.05), including full irrigation and drought stress treatments (**Figure 6**). Under all water treatments (full irrigation and drought stress), from cluster I to cluster III, the maize yield increased significantly, the above-ground biomass at silking significantly decreased. The harvest index significantly increased (**Figure 5**). This finding demonstrates that the higher yield of modern cultivars under drought stress, particularly under both moderate and intense conditions, is primarily achieved by maintaining a higher harvest index. The observed trend can be linked to superior traits in modern hybrids, notably reduced ASI and a lower incidence of empty ears under drought conditions relative to older cultivars (Sun et al., 2017; Campos et al., 2004; Liu et al., 2023), which reduces the kernel number per ear (Edreira et al., 2011; Oury et al., 2016a; Adotey et al., 2021; Liu et al., 2022) and drought-induced yield loss in older maize cultivars stems from several factors: reduced pollen quantity and viability (Liu et al., 2023), suppressed silk and ovary growth, and impaired silk receptivity (Oury et al., 2016a, Oury et al., 2016b; Dresselhaus and Franklin-Tong et al., 2013; Wilhelm et al., 1999; Alam et al., 2017).

Root systems serve as the primary route for soil resource uptake and transport, and their function is closely related to root size and morphology (Jackson et al., 2000; Fan et al., 2016). The root system absorbs photoassimilates, which helps plants withstand poor growing environments (Bañoc et al., 2000). Plants fine-tune their root systems in response to environmental stress to secure water, and this root development fundamentally shapes their stress sensitivity and tolerance (Smucker, 1993). In this study, maize root weight decreased significantly under moderate drought stress (**Figure 5**). With the year release, maize root weight decreased significantly under full irrigation and moderate drought stress (*p* < 0.05). The strong drought resistance of modern maize cultivars may be attributed to their smaller plant and root size, which leads to lower consumption of photosynthetic substances (Liu et al., 2004). Greater plant and root biomass require more energy for maintenance, which is disadvantageous in drought conditions. Under drought stress, reductions in plant and leaf area help decrease transpiration rates, thereby enhancing drought resistance (Messina et al., 2015; Liu et al., 2023).

Additionally, in the 0-10 cm soil layer, the root distribution ratios of XY335 and JNK728 increased under drought stress, whereas those of DY13, YD13, and ZD958 decreased compared with full irrigation treatments. In the 40-60 cm soil layer, with cultivar advancement, XY335 and JNK728 exhibited a higher root distribution ratio than DY13, YD13, and ZD958 under moderate drought stress (**Figure 7**). Under drought stress conditions, elongation of the maize root systems facilitates deeper soil water absorption, thereby enhancing drought resistance (Blum and Johnson, 1993).

Drought significantly affects photosynthesis, as well as dry matter accumulation and distribution, in maize (Xu et al., 2025; Liu et al., 2023). In this study, we found that: under full irrigation, net photosynthetic rates of Cluster I, II, and III were significantly higher than that under moderate and intense drought conditions. However, there was no significant difference in net photosynthetic rate between moderate and severe drought conditions (**Figure 10**). with the year release, a significant linear increase in maize net photosynthetic rate was observed under fully irrigated treatments (*p* < 0.05), but no significant relationships were found under moderate and intense drought conditions (**Figure 9**). This suggests that the enhanced drought tolerance of modern maize cultivars is not attributable to improvements in photosynthetic rate. Under drought conditions, the higher yields of modern cultivars may be primarily due to their shorter anthesis-silking interval (ASI), better pollination efficiency, and consequently, higher numbers of ears per hectare, kernels per ear (Liu et al., 2023) and harvest index.

### 4.3 Screening the drought resistance index of maize

Drought tolerance in plants is typically evaluated based on phenotypic, physiological, and biochemical responses, with phenotypic traits being particularly crucial for breeding drought-resistant cultivars (Ribaut and Ragot, 2007; Reynolds and Langridge, 2016). Identifying sensitive phenotypic indicators of drought resistance is essential for developing improved maize cultivars. In this study, varying correlation coefficients were observed between maize yield and phenotypic traits, ranked in descending order as follows: ear number per hectare (X2) > tasseling-to-silking interval (X1) > ear kernel number (X3) > ear diameter (X10) > SPAD at 30 days after silking (X8) > ear length (X9) > SPAD at silking (X7) > ear height (X13) > plant height (X12) (**Table 3**). Through stepwise regression analysis, four indicators significantly correlated with maize drought resistance were screened out which were ear number per hectare (X2), ear height (X13), tasseling to silking interval (X1), and ear length (X9). The highest correlations were observed between maize yield and ear number per hectare (X2), ear height (X13), tasseling to silking interval (X1), and ear length (X9), indicating that drought stress primarily extended the tasseling-to-silking interval, severely impacting maize pollination. This consequently reduced both the number of ears and the kernel count per ear (Liu et al., 2023; Kadam et al., 2014; Maman et al., 2004; Mbanyele et al., 2021). The superior drought resistance of modern maize cultivars was largely attributed to a shorter tasseling-to-silking interval under drought stress, resulting in a higher number of ears per hectare and a higher kernel count per ear (Liu et al., 2023). Additionally, drought stress accelerated leaf senescence, leading to decreased leaf SPAD values (Smit and Singels, 2006), and negatively affected plant growth, reducing both plant height and ear height (Sari-Gorla et al., 1999) and ear length (Khatibi et al., 2023).

### 4.4 Limitations of the study

Regarding the limitations of this study, we will address the following four aspects:

- Experimental duration: This study involved only two growing seasons (2018–2019);
- Experimental location: The study was conducted at a single site in Hengshui, Hebei, primarily because this station is equipped with rain-out shelter facilities suitable for this experiment;
- Cultivar selection: Nine maize cultivars from different eras were selected as experimental materials. Although these cultivars were widely promoted and planted in their respective eras, they may not fully represent all breeding eras. Nevertheless, they are representative of typical maize cultivars from their corresponding time periods;
- Root data: Root data were obtained for only five cultivars and two water treatments (full irrigation, moderate drought). The main reason is the intensive workload associated with root sampling. Therefore, five cultivars with contrasting drought tolerance were selected. Among the three water treatments (full irrigation, moderate drought, and severe drought), full irrigation and moderate drought were chosen because the yield difference between these two treatments is relatively large, which facilitates better observation of root responses.

## 5 Conclusion

To investigate the reasons for the enhanced drought tolerance of modern cultivars and to screen drought tolerance indicators, this study characterized grain yield in a historical panel of maize cultivars (1970s–2010s) under well-watered and drought-stressed conditions. A significant linear increase in maize yield with year of release (*p* < 0.05) was observed, with modern cultivars exhibiting the highest yields under both irrigation and drought conditions, demonstrating superior drought resistance. The primary factors influencing drought resistance and maize yield during the study period included a significant increase in the harvest index, especially under both moderate and intense drought stress. Additionally, reductions in root size and increases in deep-soil root distribution in modern cultivars were identified as key contributors to enhanced drought resistance. Screening of the drought resistance index revealed that drought stress had the greatest impact on ear number per hectare (X2), ear height (X13), tasseling to silking interval (X1), and ear length (X9).

## Data Availability

The original contributions presented in the study are included in the article/supplementary material. Further inquiries can be directed to the corresponding authors.
